# Aldoxime Metabolism Is Linked to Phenylpropanoid Production in *Camelina sativa*

**DOI:** 10.3389/fpls.2020.00017

**Published:** 2020-02-05

**Authors:** Dingpeng Zhang, Yeong Hun Song, Ru Dai, Tong Geon Lee, Jeongim Kim

**Affiliations:** ^1^ Horticultural Sciences Department, University of Florida, Gainesville, FL, United States; ^2^ Gulf Coast Research and Education Center, University of Florida, Wimauma, FL, United States; ^3^ Plant Molecular and Cellular Biology Graduate Program, University of Florida, Gainesville, FL, United States

**Keywords:** *Camelina sativa*, aldoxime, phenylpropanoids, auxin, PAL degradation

## Abstract

Plants produce diverse secondary metabolites. Although each metabolite is made through its respective biosynthetic pathway, plants coordinate multiple biosynthetic pathways simultaneously. One example is an interaction between glucosinolate and phenylpropanoid pathways in *Arabidopsis thaliana*. Glucosinolates are defense compounds made primarily from methionine and tryptophan, while phenylpropanoids are made from phenylalanine. Recent studies have shown that the accumulation of glucosinolate intermediate such as indole-3-acetaldoxime (IAOx) or its derivatives represses phenylpropanoid production *via* the degradation of phenylalanine ammonia lyase (PAL) functioning at the entry point of the phenylpropanoid pathway. Given that IAOx is a precursor of other bioactive compounds other than glucosinolates and that the phenylpropanoid pathway is present in most plants, we hypothesized that this interaction is relevant to other species. *Camelina sativa* is an oil crop and produces camalexin from IAOx. We enhanced IAOx production in Camelina by overexpressing *Arabidopsis CYP79B2* which encodes an IAOx-producing enzyme. The overexpression of *AtCYP79B2* results in increased auxin content and its associated morphological phenotypes in Camelina but indole glucosinolates were not detected in Camelina wild type as well as the overexpression lines. However, phenylpropanoid contents were reduced in *AtCYP79B2* overexpression lines suggesting a link between aldoxime metabolism and phenylpropanoid production. Interestingly, the expression of *PALs* was not affected in the overexpression lines although PAL activity was reduced. To test if PAL degradation is involved in the crosstalk, we identified F-box genes functioning in PAL degradation through a phylogenetic study. A total of 459 transcript models encoding kelch-motifs were identified from the *Camelina sativa* database. Among them, the expression of *CsKFBs* involved in PAL degradation is up-regulated in the transgenic lines. Our results suggest a link between aldoxime metabolism and phenylpropanoid production in Camelina and that the molecular mechanism behind the crosstalk is conserved in Arabidopsis and Camelina.

## Introduction

As sessile organisms, plants produce diverse secondary metabolites which play crucial roles in their adaptation to surrounding conditions. Although each metabolite is produced through its respective biosynthetic pathway, plants orchestrate multiple biosynthetic pathways simultaneously. Recent studies have shown the influence of glucosinolate intermediates on phenylpropanoid production (Hemm et al., 2003; Kim et al., 2015; Kim et al., 2019).

Glucosinolates are defense compounds and over 100 kinds of glucosinolates have been found in plants (Agerbirk and Olsen, 2012). Phenylpropanoids such as lignin and flavonoids are made from phenylalanine through the phenylpropanoid pathway and are involved in diverse aspects of plant growth and development (Vogt, 2010; Fraser and Chapple, 2011). Although phenylpropanoids and glucosinolates are produced through their own biosynthetic pathways, a link between the two pathways was identified from Arabidopsis studies.

Arabidopsis mutants with defects in the formation of the core structure of glucosinolates show defects in both phenylpropanoid production and glucosinolate biosynthesis (Hemm et al., 2003; Kim et al., 2015). For instance, Arabidopsis *ref5* and *ref2* mutants were originally isolated due to their phenylpropanoid deficiency phenotypes (Ruegger and Chapple, 2001). However, genetic mapping studies revealed that *ref5* and *ref2* have mutations in CYP83B1 and CYP83A1, respectively (Hemm et al., 2003; Kim et al., 2015). Both CYP83A1 and CYP83B1 are involved in the oxidation of aldoximes, which is required for the core structure of glucosinolates (Naur et al., 2003). Although both genes have broad substrate specificities toward aldoximes, CYP83A1 functions mainly in aliphatic glucosinolate biosynthesis whereas CYP83B1 has higher activity toward indole-3-acetaldoxime (IAOx). Further biochemical studies have revealed that the accumulation of aldoximes or their derivatives, rather than a deficiency of glucosinolates or the accumulation of IAA, is responsible for the repression of phenylpropanoid production in *ref5* and *ref2* (Hemm et al., 2003; Kim et al., 2015). Studies with *ref5* suppressor revealed that Arabidopsis mediator subunit 5 (MED5) is involved in the link between the glucosinolate and phenylpropanoid biosynthesis pathways. The steady state phenylalanine pools in *ref5* mutants were higher than that in the wild type suggesting that the inhibition may occur at or downstream of PAL in the phenylpropanoid biosynthetsis pathway (Kim et al., 2015). A transcriptome study with a set of glucosinolate-deficient mutants identified that there are MED5-dependent and MED5–independent mechanisms behind the crosstalk, and that the increased levels of aldoximes up-regulate kelch-domain containing F-box genes functioning in the ubiquitination of PALs (Kim et al., 2019).

**Figure 1:**
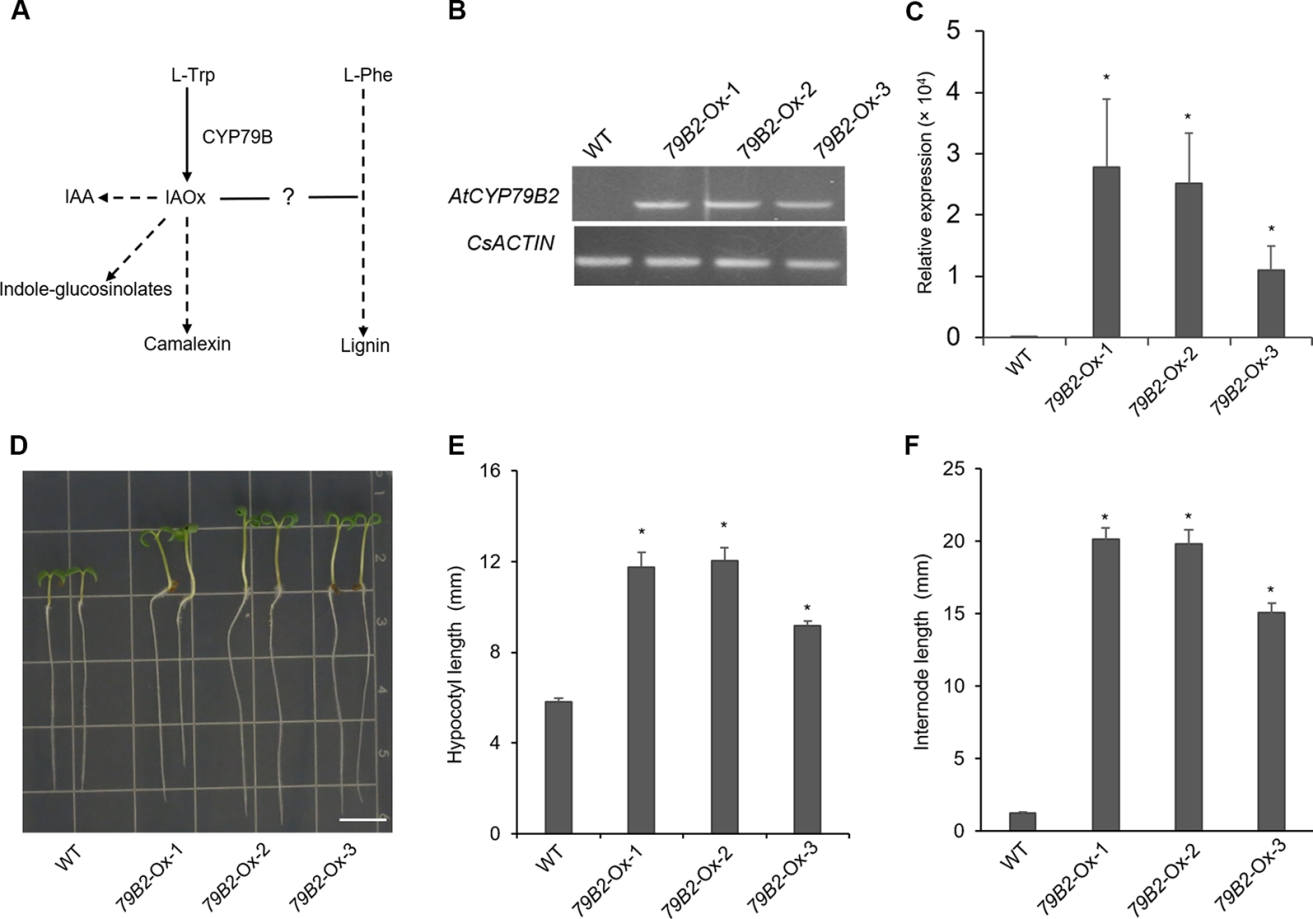
Overexpression of *AtCYP79B2* in Camelina results in characteristic morphologicalphenotypes. **(A)** A proposed link between IAOx metabolism and the phenylpropanoid pathway in Camelina. **(B**, **C)** Expression of *AtCYP79B2* in three independent transgenic lines, *79B2-*Ox-1, *79B2-*Ox-2, and *79B2-*Ox-3, was measured by semiquantitative RT-PCR **(B)** or quantitative RT-PCR **(C)**. Total RNA was prepared from 14-day-old Camelina leaves grown on soil and *CsACTIN* was used as control. In **(C)**, data represent mean ± standard error (SE) from four independent biological replicates. **(D)** Representative 7-day-old Camelina plants grown on MS plate were photographed. Bar = 1 cm. **(E**, **F)** Hypocotyl length of 7-day-old seedlings **(E)** and the internode length between the second leaf and the third leaf **(F)** were measured. 14-day-old plants grown on soil were used for the internode measurement. In **(E**, **F)**, data represent mean ± standard error (SE) (n = 16). * represents *P* < 0.05 (two-tailed student’s t-test) when compared with wild type.

The aldoximes are made from various amino acids by the cytochrome P450 monooxygenase 79 family members (CYP79) (Irmisch et al., 2013; Irmisch et al., 2015; Luck et al., 2017; Bhalla et al., 2018; Sørensen et al., 2018; Dhandapani et al., 2019). *Arabidopsis thaliana* has five functional CYP79s. CYP79F1 and CYP79F2 convert homomethionine to aliphatic aldoximes and CYP79A2 produces phenylacetaldoxime from phenylalanine (Wittstock and Halkier, 2000; Hansen et al., 2001; Chen et al., 2003). CYP79B2 and CYP79B3 function redundantly to convert tryptophan to IAOx (Mikkelsen et al., 2000; Zhao et al., 2002). In particular, IAOx is a precursor of indole-glucosinolates, IAA, and camalexin (Zhao et al., 2002; Sugawara et al., 2009; Klein et al., 2013) (**Figure 1A**), therefore overexpression of *AtCYP79B2* increases the production of IAA and indole glucosionolates (Zhao et al., 2002). Since aldoximes are precursors of various metabolites in addition to glucosinolates (Irmisch et al., 2013; Irmisch et al., 2015; Luck et al., 2017; Bhalla et al., 2018; Sørensen et al., 2018; Dhandapani et al., 2019) and the phenylpropanoid pathway is conserved in most plants, it is possible that a similar interaction occurs in other plant species. This study aims to elucidate a connection between IAOx metabolism and the phenylpropanoid pathway in *Camelina sativa* (**Figure 1A**).

*Camelina sativa* is an annual Brassicaceae oilseed crop. Due to its high levels of oil and unique oil composition with over 90% unsaturated fatty acid in its seeds, it is a valuable industrial oil crop for making jet fuel and biodiesel (Kagale et al., 2014). In addition, its seed meal is used as animal feed because of its residual essential fatty acids (Malik et al., 2018). Camelina produces glucosinolates and phenylpropanoids including sinapine (Russo and Reggiani, 2012). Since glucosinolates and sinapine limit the use of Camelina flours in animal diet due to their antinutritional properties (Russo and Reggiani, 2012), it is of great interest to manipulate these secondary metabolites. Given that *C. sativa* produces glucosinolates and camalexin from aldoximes (Browne et al., 1991; Klein et al., 2013), it is likely that aldoxime metabolism and the phenylpropanoid pathway coexist in *C. sativa.* To examine the impact of altered aldoxime metabolism on the phenylpropanoid pathway in *C. sativa,* we enhanced the IAOx production in *C. sativa* by overexpressing Arabidopsis *CYP79B2* and evaluated its influence on plant growth and phenylpropanoid production. Our data suggest that the links between indole aldoxime metabolism and phenylpropanoid production found in Arabidopsis also occur in other Brassicaceae such as *C. sativa*.

## Materials and Methods

### Plant Materials and Growth Conditions

*Camelina sativa* cv. Celine and *Arabidopsis thaliana* Col-0 were used as wild-type plants. Plants were grown at 22°C ± 1°C with 16-h light/8-h dark photoperiod. For seedlings grown on MS plates, the seeds were sterilized with 20% (v/v) bleach containing 0.005% triton X-100 for 10 min. After being washed with water multiple times, the seeds were cold-treated at 4°C for 3 days, and then were planted on MS medium containing 2% sucrose and 1.2% agar. For soil-grown plants, the seeds were directly planted on soil after 3 days of cold treatment at 4°C.

### Generation of Transgenic Plants Overexpressing *AtCYP79B2* and *CsKFB50*

For Camelina *AtCYP79B2* overexpression lines, the published 995-AtCYP79B2 binary vector that carries *AtCYP79B2* under the CaMV 35S promoter was used for Camelina transformation (Kim et al., 2015). To generate a *Camelina sativa Csa05g091560* overexpression construct, the open reading frame of *Csa05g091560* was amplified by PCR using the primer pair of P19 and P20 and cDNA prepared from 6-day-old seedlings (**Table S4**). The amplified PCR product was cloned into a Gateway entry vector pCC1155, a modified version of pDONR221, and the resulting *Csa05g091560* entry vector was recombined with destination vector pCC0995 (Weng et al., 2010), generating the 35S:Csa05g091560 construct. The intact ORF of Csa05g091560 in the 35S:Csa05g091560 construct was confirmed by sequencing before it was introduced into plants.

The constructs were introduced into Arabidopsis or Camelina plants by Agrobacterium*-*mediated flower dipping methods as previously described (Lu and Kang, 2008). The cultured *Agrobacterium tumefaciens* (strain GV3101) harboring the constructs was suspended in 5% sucrose containing 0.01% (v/v) Silwet L-77 (PhytoTech, S7777). The flowers of mature plants were dipped in the Agrobacterium solution for 30 s, and then the plants were kept for 24 h in the dark. To screen transformants, 10-day-old plants were sprayed with 0.2% Basta (Rely 280, BASF, NJ) and surviving T1 lines were confirmed with the expression of *AtCYP79B2* or *Csa05g091560*.

### Measurement of Hypocotyl and Internode Length

Seven-day-old T4 generation Camelina seedlings grown on MS plates were used for hypocotyl measurement. All of the transgenic lines used for phenotyping were confirmed for the presence of constructs either by spraying Basta or PCR for BAR gene in the vector (Weng et al., 2010).

Two-week-old soil-grown Camelina plants were used for the internode measurement. Sixteen samples (N = 16) were measured for both experiments and significance was tested with a two-tailed student’s t-test.

### Metabolite Analysis

For phenylpropanoid analyses, fresh samples were frozen in liquid nitrogen and stored at −80°C until extraction. Metabolites were extracted with 50% (v/v) methanol at 65°C for 1 h. After centrifugation at 10,000 x *g* for 10 min, the supernatant was collected for HPLC analysis. Ten μl of extracts was analyzed on an UltiMate 3000 HPLC system (ThermoFisher Scientific, MA) equipped with an autosampler cooled to 10°C and a diode array detector (DAD). The compounds were separated on an Acclaim™ 120 C18 column (75 mm × 3 mm; 2.2 μm) coupled with a C18 guard column (10 mm × 3 mm; 5 μm) (ThermoFisher Scientific, MA), with mobile phases consisting of solvent A; 0.1% formic acid (v/v) in water and solvent B; acetonitrile, and the following gradient: 5%–20% increase of solvent B at 0–6 min, 20%–95% increase of solvent B at 6–8.5 min, and 95% solvent B at 8.5–10 min. The column was equilibrated for 5 min between injections. The flow rate was 0.7 ml min^-1^ and the column temperature was 40°C. The contents of chlorogenic acid and sinapoylmalate were quantified based on the peak area at 325 and 330 nm, respectively, and chlorogenic acid (Sigma, MO, C3878) and sinapic acid (Sigma, MO, D7927) were used for the external standards. For glucosinolate analyses, frozen plant samples were extracted with 50% (v/v) methanol at 65°C for one and a half hours. The relative levels of indole-3-ylmethyl glucosinolate (I3M) were quantified based on the peak area at 229 nm (Lee et al., 2012). Soluble metabolite analyses were performed with three biological replicates and statistical significance was tested with a two-tailed student’s t-test.

### Purification of IAA

IAA analysis was performed as described by Novák et al. (2012) with slight modifications. Twenty mg of each sample was homogenized and extracted in 1 ml of sodium phosphate buffer (50 mM, pH 7.0, 4°C) containing 0.1% diethyldithiocarbamic acid sodium salt. [^13^C_6_]-IAA (5 pmol per sample) was added as an internal standard. The plant extracts were incubated at 4°C for 60 min with continuous shaking and then centrifuged at 16,300 x *g* at 4°C for 15 min. After centrifugation, the sample pH was adjusted to pH 2.7 with concentrated hydrochloric acid, and the extracts were purified using solid-phase extraction on Oasis™ HLB columns (1 ml/30 mg, Waters, MA). The columns were conditioned with 1 ml methanol and 1 ml water, and equilibrated with 1 ml sodium phosphate buffer (pH 2.7). After sample application, the column was washed with 4 ml of 5% methanol in water and eluted with 4 ml of 80% methanol. The eluate was evaporated to dryness *in vacuo* and stored at -20°C until further analysis.

### Quantitative Analysis of IAA

All samples were analyzed by LC-MRM-MS (liquid chromatography multiple reaction monitoring mass spectrometry). The evaporated samples were dissolved in 100 µl of mobile phase (35% methanol containing 0.1% formic acid) prior to mass analysis using a TSQ Altis Triple Quadrupole MS/MS system with ion funnel connected to a Vanquish Horizon ultra-high performance liquid chromatography (UHPLC) system equipped with an Accucore C18 column, 3 × 50 mm, 2.6 µm (Thermo Scientific, MA). MRM parameters of IAA and labeled IAA (precursor *m/z*, fragment *m/z*, radio frequency (RF) lens, and collision energy) were optimized on the TSQ Altis Triple Quadrupole MS using direct infusion of the authentic standards. A binary gradient of 0.1% formic acid in water and 0.1% formic acid in methanol was used as mobile phases A and B, respectively. The gradient profile was: 35% solvent B from 0–5 min and 35% to 95% solvent B from 5–6 min, and the flow rate was 0.25 ml min^-1^. Afterwards, the column was washed with 95% methanol for 5 min and re-equilibrated to initial conditions for 5 min. The mass spectrometer conditions were as follows: the spray voltage was applied at 3,200 V in the positive mode, and sheath gas, aux gas, and sweep gas were set at 50, 10, 0 (arb unit), respectively. Ion transfer tube and vaporizer temperatures were set at 200°C and 40°C, respectively. For MRM monitoring, both Q1 and Q3 resolutions were set at 0.7 FWHM with CID gas at 1.5 mTorr. The scan cycle time was at 0.8 s. Quantification was obtained by MRM of the precursor ([M+H]^+^) and the appropriate product ion using optimal RF value and collision energy for the various diagnostic transitions shown in **Table S1**. The analysis was done with four replicates and statistical significance was tested with a two-tailed student’s t-test.

### PAL Activity

The PAL activity assay was performed as described with some modifications (Kim et al., 2015). Crude proteins were extracted with protein extraction buffer (0.1 M Tris (pH 8.3), 10% glycerol, and 5 mM DTT) After extraction, protein content was measured by Bradford protein assay with Bradford reagent (Sigma, MO, B6916) following the manufacturer’s protocol. Bovine serum albumin (BSA, ThermoFisher Scientific, MA) was used as a standard. For the PAL activity assay, 150 μl of protein extract was mixed with 4 mM phenylalanine in a total of 500 μl reaction solution (0.1 M Tris (pH 8.3), 10% glycerol, 5 mM DTT) and incubated at 37°C for 90 min. The reaction was stopped by adding 50 μl of acetic acid and the reaction product was extracted with 750 μl of ethylacetate. The mixture was centrifuged at 10,000 x *g* for 5 min and 600 μl of supernatant was transferred into microcentrifuge tubes and dried in Vacufuge plus (Eppendorf, NY). The final extract was dissolved in 90 μl of 50% methanol and analyzed using HPLC. PAL activity assays were performed with three biological replicates and statistical significance was tested with a two-tailed student’s t-test.

### RNA Extraction and Quantitative RT-PCR

Total RNA was extracted with RNeasy Mini Kit (QIAGEN, MD, 74904). Three μg of total RNA was treated with DNase (Invitrogen, MA, AM1907) following the manufacturer’s protocol, and then 1 μg of RNA was used for the first-strand cDNA synthesis using reverse transcription kit (ThermoFisher Scientific, MA 4368814). Quantitative PCR was performed with SYBR Green PCR master mix (ThermoFisher Scientific, MA) in StepOnePlus Real-Time PCR system. The specific primers are listed in **Table S4**. The following primers were used: AtCYP79B2, P1 and P2; AtTUBULIN, P47 and P48; CsACTIN, P3 and P4; CsKFB1-like, P98 and P99; CsKFB20-like, P9 and P10; CsKFB39-like, P100 and P101; CsKFB50-like, P102 and P103; CsPAL-like1, P121 and P122; CsPAL-like2, P125 and P126. For semiquantitative RT-PCR, 0.5 μl cDNA was used as a template. PCR reactions were performed in a 20 μl volume mix containing Taq 2x Master Mix (NEB, M0270S) and primers (**Table S4**). P1 and P2 were for *AtCYP79B2* while P3 and P4 were for *CSACTIN*.

### Identification of *C. sativa* Kelch-Motif Orthologs Using Hidden Markov Models

The *C. sativa* annotated protein dataset was obtained from NCBI (https://www.ncbi.nlm.nih.gov/genome/annotation_euk/Camelina_sativa/101).Putative orthologs of Arabidopsis genes were identified by using a hidden Markov model search (HMMER3.1b2;http://hmmer.org). The seed alignment model(PF01344) from Pfam (https://pfam.xfam.org; data access on Apr. 19, 2018) was used to identify canonical proteins of *C. sativa*. 10^-1^ *e*-value cutoff was applied. No manual curation of *C. sativa* sequences and screening of conserved motifs outside the kelch-motif were performed. In order to increase confidence that true orthologs were detected, a reciprocal BLAST search of the predicted *C. sativa* proteomes was performed using full-length Arabidopsis sequences. BLAST searches were performed using tools provided at NCBI (https://www.ncbi.nlm.nih.gov), and TAIR (http://www.arabidopsis.org). All the *C. sativa* proteins identified as matching Arabidopsis kelch-motif proteins are detected with an e-value of 0.0 by BLAST. Alignments were performed using the MUSCLE tool (muscle 3.8.31; Edgar, 2004 for the Linux platform. Maximum likelihood (ML) phylogenetic analysis of the protein sequence alignment was performed using RAxMLHPC-PTHREADS-SSE3 (version 8.2.3; Stamatakis, 2014 for the Linux platform using the VT model of amino acid substitutions and gamma distribution parameters estimated by the software. One thousand bootstraps were performed. Phylogenetic trees were visualized and edited in Geneious R10.2.2 (http://www.biomatters.com). The ML tree annotated by RAxML was provided in Newick format.

### Identification of *C. sativa* CYP79B2 and KFB50 Sequences

For CYP79B2 and KFB50 amino acid alignments, their putative homologies in *C.sativa* were identified by using the whole sequence of Arabidopsis proteins with NCBI (https://www.ncbi.nlm.nih.gov) blast tools. The alignments were constructed in T-coffee website (http://tcoffee.crg.cat/apps/tcoffee/index.html), and were visualized in The Sequence Manipulation Suite (https://www.bioinformatics.org/sms/index.html). For CYP79B2 phylogenetic analysis of the protein sequence, the ML method and Poisson model were used in MEGA-X based on ClustalW alignment. Bootstrap with 1,000 replicates was performed to show the tree topology.

## Results

### The Overexpression of *AtCYP79B2* Increases Auxin Content and Affects Plant Growth and Development in *C. sativa*

CYP79B family converts tryptophan to IAOx in Arabidopsis. Seven CYP79B homologs that are highly similar to those of *Arabidopsis thaliana* CYP79B2 (AtCYP79B2) and its paralog AtCYP79B3 were identified using the *Camalina sativa* protein database (**Table 1**). Four of them (Csa10g002730, Csa12g003200, Csa12g002890, and Csa11g003080) show over 96% amino acid sequence identity with AtCYP79B2 whereas the other three homologs (Csa7g052430, Csa16g044070, and Csa9g086670) are more related to AtCYP79B3 (**Table 1**). Considering their conserved domains and high sequence homology (**Figure S1**), it is likely that CsCYP79Bs have biochemical activities similar to that of AtCYP79Bs. To increase IAOx production in *C. sativa*, Camelina transgenic lines overexpressing *AtCYP79B2* were generated (**Figure 1**). Among a series of *AtCYP79B2* overexpression lines, three independent lines showing high expression of *AtCYP79B2* were selected for further study (**Figures 1B, C**).

**Table 1:** Protein sequence identity of Camelina CYP79Bs compared with Arabidopsis CYP79Bs.

Gene name	Chromosome	Identity (%)	
		AtCYP79B2	AtCYP79B3
Csa10g002730	10	97	85
Csa12g003200	12	96	85
Csa12g002890	12	96	84
Csa11g003080	11	96	84
Csa7g052430	7	84	93
Csa16g044070	16	85	93
Csa9g086670	9	78	86

**Figure 2:**
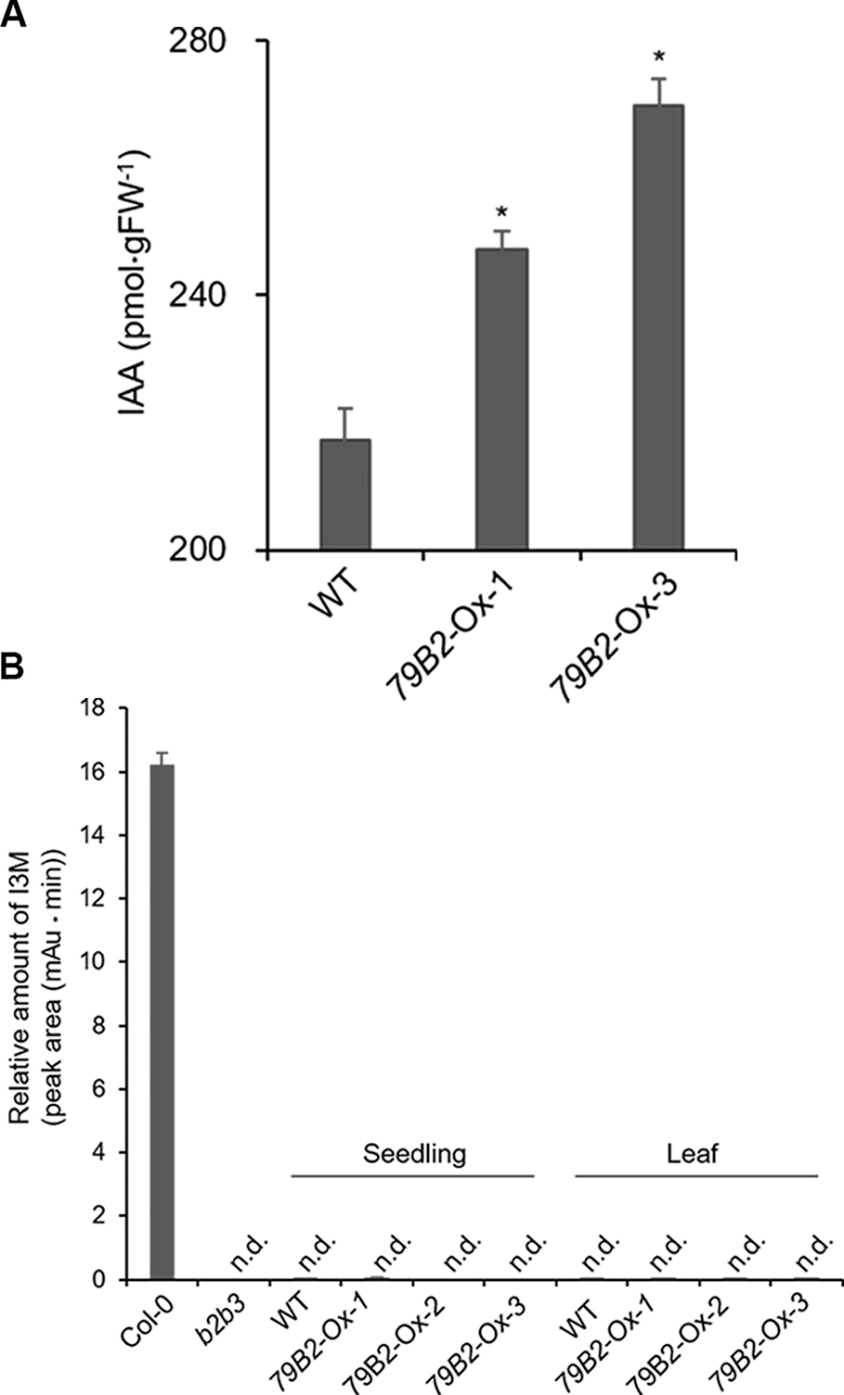
The Levels of IAA are Increased but Indole Glucosiniolate is Deficient in Camelina Transgenic Plants Overexpressing *AtCYP79B2*. **(A)** Free IAA contents in the Camelina overexpression lines and wild type. Six-day-old seedlings grown on MS medium were collected for IAA measurement. Data represent mean ± SE of four independent experiments. * represents *P* < 0.05 (two-tailed student’s t-test) when compared with wild type. **(B)** Relative levels of indole-3-ylmethyl glucosinolate (I3M) in *C. sativa* and *A. thaliana*. The levels of I3M were measured from 6-day-old Camelina seedlings grown in MS medium or leaves from 8-week-old plants grown on soil. *Arabidopsis* Col-0 and *cyp79b2 cyp79b3* (*b2b3*) were used as positive and negative controls. Relative contents of I3M are shown based on peak areas of HPLC chromatograms at the retention time where I3M in Col-0 was eluted. Data represent mean ± SE of three independent experiments. n.d., not detected.

Interestingly, Camelina plants overexpressing *AtCYP79B2* display characteristic morphological phenotypes such as curled down cotyledons and elongated hypocotyls and internodes that were previously observed in plants containing increased levels of auxin (**Figures 1D–F** and **Figure S2**, Delarue et al., 1998; Zhao et al., 2002; Kim et al., 2007; Kim et al., 2015). Indeed, the level of IAA in the transgenic lines is higher than that of wild type plants (**Figure 2A**). It thus appears that the overproduced IAOx in Camelina is redirected to the production of IAA as observed in Arabidopsis.

### The Increased IAOx Production Negatively Impacts the Phenylpropanoid Production in *C. Sativa*

**Figure 3:**
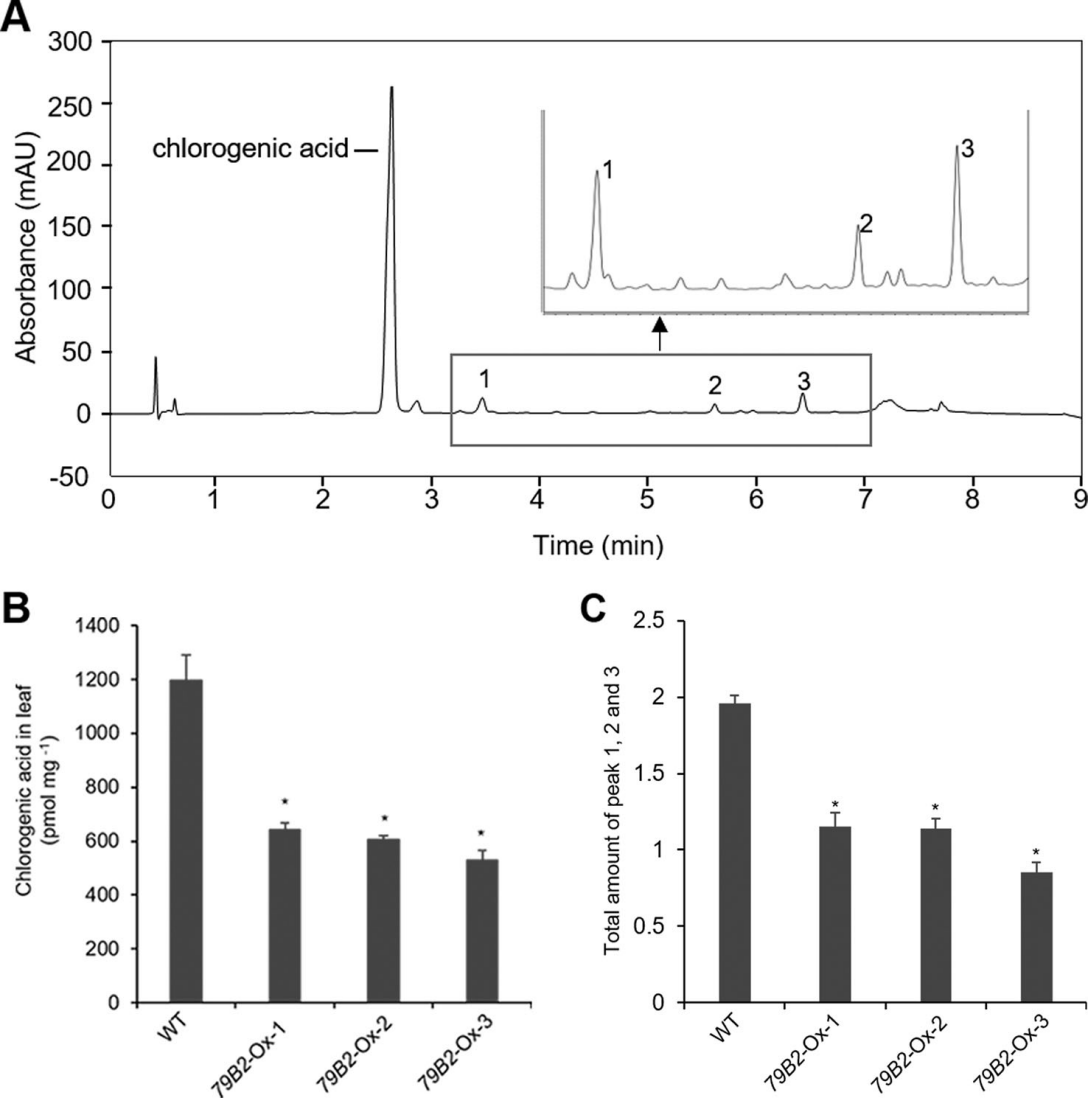
Overexpression of *AtCYP79B2* inhibits phenylpropanoid production in Camelina.Representative chromatogram of soluble metabolites in wild-type Camelina leaves is shown **(A)**. The extract gave four recognizable peaks at 325 nm and chlorogenic acid is a major peak. The inset shows three peaks eluted between 3 and 7 min. Chlorogenic acid contents **(B)** and the total sum of peak 1, 2, and 3 **(C)** of the *AtCYP79* overexpression lines compared with wild type are shown. Leaf samples were collected from 2-week-old soil grown plants. The total amount of peak 1 to 3 was calculated based on the peak area of each peak. In **(B)** and **(C)**, data represent mean ± SE of three independent experiments. * represents *P* < 0.05 (two-tailed student’s t-test) when compared with wild type.

**Figure 4:**
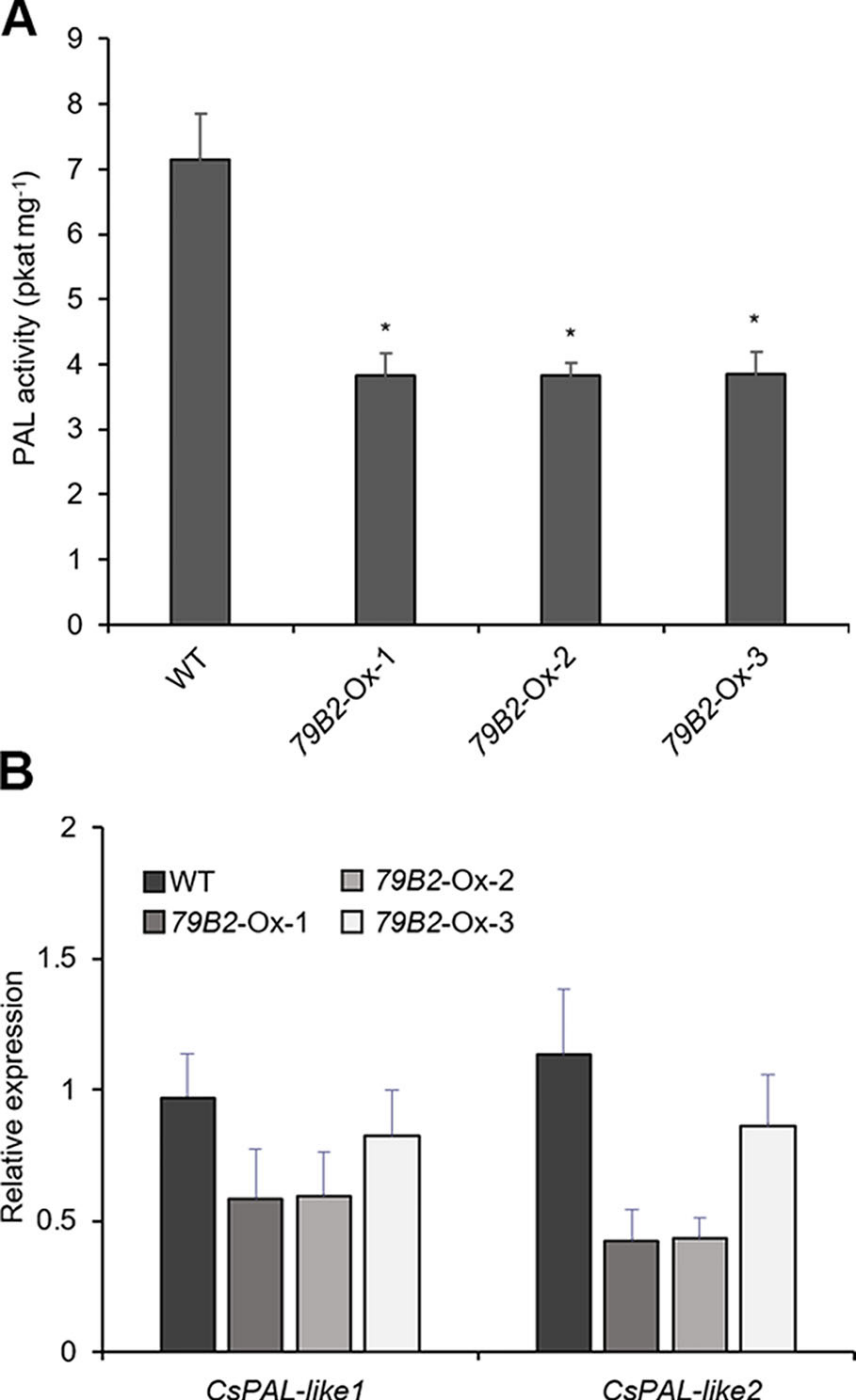
Phenylalanine ammonia lyase (PAL) activity is reduced in *AtCYP79B2* overexpression lines. **(A)** PAL activities were measured using crude extracts of leaves from 2-week-old soil grown Camelina plants. **(B)** Transcript abundance of Camelina PALs in transgenic lines and wild type was measured with quantitative RT-PCR. CsPAL-like1 represents expression of six genes (Csa06g039710, Csa05g016810, Csa04g050600, Csa06g027730, Csa04g039260, and Csa09g061550) which are Arabidopsis PAL1 and PAL2 homologs. CsPAL-like2 represents expression of Csa01g011180 and Csa15g012820, which are Arabidopsis PAL4 homologs. In **(A)** and **(B)**, data represent mean ± SE of three independent experiments. * represents *P* < 0.05 (two-tailed student’s t-test) when compared with wild type.

Arabidopsis plants overexpressing *CYP79B* contained increased indole glucosinolates (Zhao et al., 2002). However, we were not able to detect indole glucosinolates in young seedlings or old leaves of both Camelina wild type and all three overexpression lines (**Figure 2B**). To test whether increased IAOx metabolism affects phenylpropanoid production in Camelina, soluble phenylpropanoid contents in seedlings, cotyledons, and mature leaves were assessed. Methanol extract from Camelina leaves gave four recognizable peaks and chlorogenic acid was a major phenylpropanoid as shown in **Figure 3A**. The levels of chlorogenic acid and the other phenolic compounds were substantially reduced in the *AtCYP79B2* overexpression lines compared to wild type (**Figure 3** and **Figure S3**). This suggests a link between aldoxime metabolism and phenylpropanoid production in Camelina. The production of chlorogenic acid from phenylalanine requires a series of enzymes. PAL is the first enzyme of the phenylpropanoid pathway that converts phenylalanine to cinnamic acid. The transgenic lines display approximately 50% of wild-type PAL activity (**Figure 4A**), which may contribute to the reduced levels of phenylpropanoids in the overexpression lines.

### The Identification of Kelch-Domain Containing F-Boxes (KFB) in *C. sativa*

PAL activity can be regulated at various levels including transcriptional and posttranslational regulations. We first assessed the levels of PAL expression in the transgenic lines. There are four PALs: PAL1, PAL2, PAL3, and PAL4 in Arabidopsis. We identified eight potential PAL homologs in Camelina. Csa06g039710, Csa05g016810, and Csa04g050600 show over 97% sequence identity with Arabidopsis PAL1, whereas Csa06g027730, Csa04g039260, and Csa09g061550 are more related to Arabidopsis PAL2. Similarly, Csa01g011180 and Csa15g012820 are Arabidopsis PAL4 homologs with 94% sequence identity. Interestingly, we were not able to find PAL3 homologs from the NCBI protein database.

Two sets of primers were designed to detect the transcript abundance of these eight genes. CsPAL-like1 detects PAL1 and PAL2 homologs (Csa06g039710, Csa05g016810, Csa04g050600, Csa06g027730, Csa04g039260, Csa09g061550) whereas CsPAL-like2 detects two PAL4 homologs (Csa01g011180, Csa15g012820). The expression levels of Cs-PAL-like1 and Cs-PAL-like2 were not statistically different in the transgenic lines from those of wild type plants (**Figure 4B**) suggesting that factors other than transcriptional regulation are suppressing PAL activity in the transgenic plants. The Arabidopsis study showed that PAL activity is affected by its stability through the 26S proteasome-mediated degradation (Zhang et al., 2013). Once PALs are ubiquitinated, the poly-ubiquitinated PALs are degraded by 26S proteasome. A specific group of kelch-domain F-Box proteins (KFBs) are involved in the PAL ubiquitination in Arabidopsis (Zhang et al., 2013; Zhang et al., 2015). These KFBs are components of ubiquitin E3 ligase, and AtKFB1, AtKFB20, AtKFB39, and AtKFB50 facilitate ubiquitination of PAL redundantly (Zhang et al., 2013; Zhang et al., 2015). In Arabidopsis, the accumulation of IAOx or its derivatives enhances the expression of these four *KFBs* and in turn, results in reduced PAL activity and phenylpropanoid production (Kim et al., 2019). When AtKFBs are disrupted in high aldoxime-containing mutants, phenylpropanoid deficiency is restored (Kim et al., 2019). Thus, we hypothesized that a similar mechanism may function in the repression of PAL in *C. sativa*.

**Figure 5:**
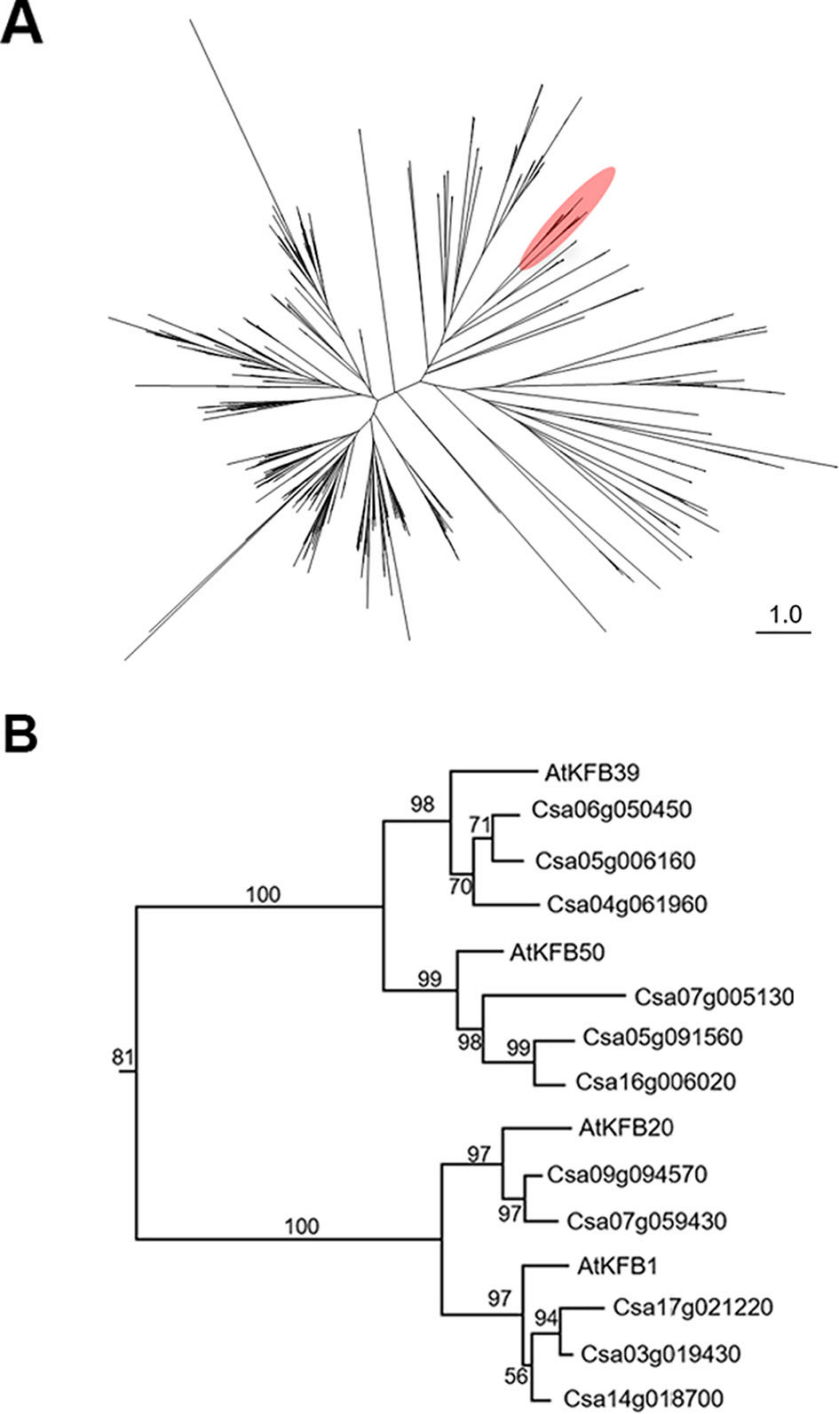
Phylogenetic analysis of Kelch-domain containing F-boxs (KFBs) in Camelina. **(A)** Maximum likelihood phylogenetic tree of *C. sativa* orthologs of four Arabidopsis genes (AtKFB1, AtKFB20, AtKFB39, AtKFB50). A radiation diagram is used to represent an unrooted maximum likelihood tree with branch lengths proportional to estimated sequence distance. A total of 459 nonredundant proteins containing kelch-motif(s) were identified. A clade including four Arabidopsis KFBs (AtKFB1, AtKFB20, AtKFB39, AtKFB50) is shown in red. **(B)** A phylogenetic tree of AtKFB1, AtKFB20, AtKFB39, and AtKFB50 homologs in Camelina shows 11 genes having high sequence similarity with AtKFBs functioning in Phenylalanine ammonia lyase (PAL) degradation.

To identify *Camelina sativa* KFB homologs, we did phylogenetic analysis for KFB homologs using the *C. sativa*-annotated protein dataset with Arabidopsis KFB1 (At1g15670), KFB20 (At1g80440), KFB39 (At2g44130), and KFB50 (At3g59940). A total of 459 transcript models containing kelch-motif(s) were identified using a Hidden Markov Model (HMM) search (**Figure 5A**). Close homologs of AtKFB1/20/39/50 were selected: Csa17g021220, Csa03g019430, and Csa14g018700 are homologs of At1g15670/KFB1; Csa09g094570, and Csa07g059430 for At1g80440/KFB20; Csa06g050450, Csa05g006160, and Csa04g061960 for At2g44130/KFB39; Csa07g005130, Csa05g091560, Csa16g006020 for At3g59940/KFB50 (**Figure 5B**). Each group of Camelina KFB homologs has 77% to 89% amino acid sequence identity with Arabidopsis KFBs (**Table S2**).

**Figure 6:**
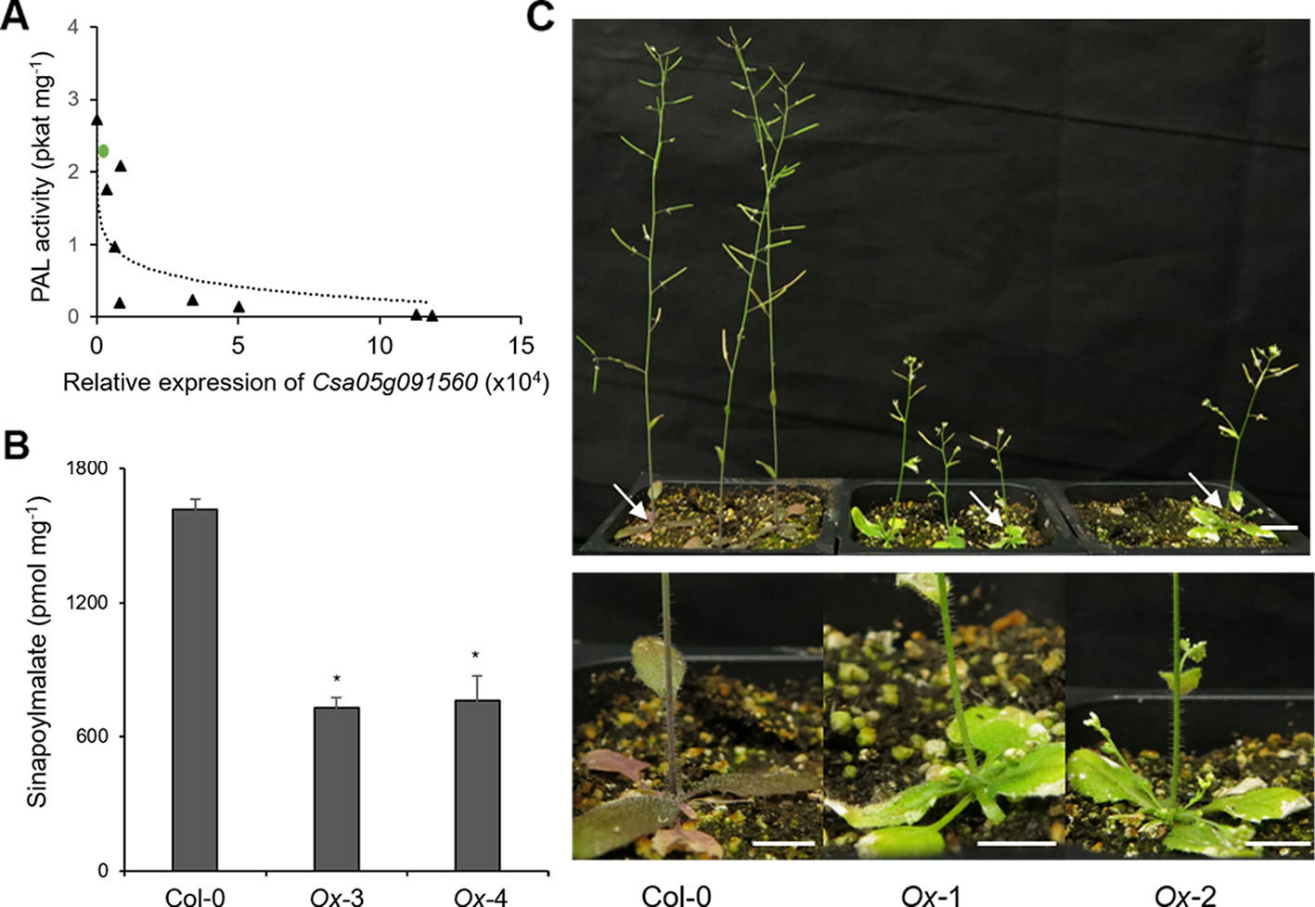
Overexpression of *Csa05g091560,* one of the *CsKFB50-like* genes,reduces Phenylalanine ammonia lyase (PAL) activity and inhibits phenylpropanoid production in Arabidopsis. **(A)** PAL activity of nine Arabidopsis transgenic lines overexpressing *Csa05g091560* is negatively correlated with the expression of *Csa05g091560*. Green circle indicates PAL activity of Arabidopsis wild type. **(B)** Sinapoylmalate contents in Arabidopsis plants overexpressing *Csa05g091560*. Soluble metabolites were extracted from 3-week-old plants grown on soil. Data represent mean ± SE of three independent experiments. * represents *p* < 0.05 (two-tailed student’s t-test) when compared with wild type. **(C)** Stunted growth and reduced anthocyanin accumulation of two transgenic lines overexpressing *Csa05g091560* are shown compared with control plants (Col-0). Close-up images of plants show anthocyanin deficiency in the transgenic lines marked with arrows. (bars = 1 cm) Photos were taken five weeks after planting.

Since CsKFB50-like genes were most abundant in our samples as shown in **Table S3**, we cloned *Csa05g091560*, one of the AtKFB50 homologs, to test its function. As shown in **Figure 6**, the expression of *Csa05g091560* in Arabidopsis was negatively correlated with PAL activity and produced lower levels of phenylpropanoids (**Figures 6A, B**). However, the transgenic lines with highly expressed *Csa05g091560* displayed stunted growth and less purple color, indicating anthocyanin deficiency (**Figure 6C**). Dwarfism and phenylpropanoid deficiency together with reduced PAL activity in the transgenic lines mimicked phenotypes in Arabidopsis *pal1/2/4* mutants and *AtKFB* overexpression lines, which suggests its possible roles on PAL degradation similar to the Arabidopsis KFBs. (Huang et al., 2010; Zhang et al., 2013; Zhang et al., 2015).

### The Expression of *CsKFBs* Functioning in PAL Degradation Is Increased in the *AtCYP79B2* Overexpression Lines

**Figure 7:**
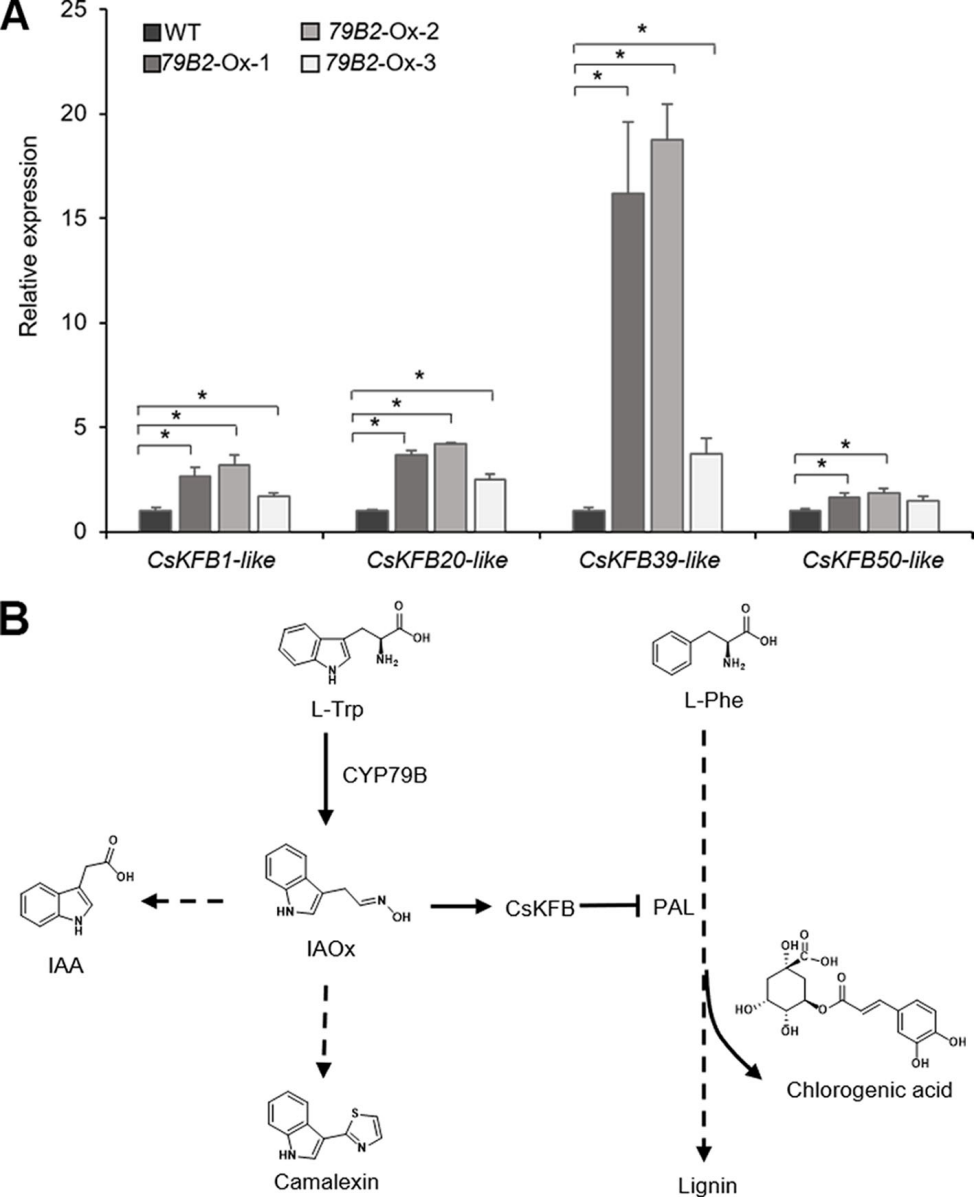
Increased expression of *CsKFBs* in the Camelina transgenic plants and a proposed model showing a link between IAOx metabolism and the phenylpropanoid pathway in Camelina. **(A)** Transcript abundance of *CsKFBs* in Camelina transgenic lines overexpressing *AtCYP79B2*. CsKFB1-like represents the expression of three KFB1 homolog genes (*Csa17g021220*, *Csa03g019430*, *Csa14g018700*). CsKFB20-like represents two CsKFB20-like genes (*Csa09g09457*0, *Csa07g059430*). CsKFB39-like and CsKFB50-like represent three CsKFB39-like genes (*Csa06g050450*, *Csa05g006160*, *Csa04g061960*) and three CsKFB50-like genes (*Csa07g005130*, *Csa05g091560*, *Csa16g006020*), respectively. Data represent mean ± SE (n = 3). * represents *P* < 0.05 (two-tailed student’s t-test) when compared with wild type. **(B)** A proposed model demonstrating that increased IAOx production influences the expression of *CsKFBs* functioning in PAL degradation, thereby affecting the phenylpropanoid production in Camelina.

To evaluate any impact of the altered aldoxime production on the expression of *CsKFBs*, we analyzed the expression levels of these *CsKFBs* in the transgenic lines. We designed four sets of primers using their conserved sequences. CsKFB1-like can detect all three AtKFB1 homologs (*Csa17g021220*, *Csa03g019430*, *Csa14g018700*). Similarly, CsKFB20-like, CsKFB39-like, and CsKFB50-like detect two AtKFB20 homologs (*Csa09g094570*, *Csa07g059430*), three AtKFB39 homologs (*Csa06g050450*, *Csa05g006160*, *Csa04g061960*), and three AtKFB50 homologs (*Csa07g005130*, *Csa05g091560*, *Csa16g006020*), respectively (**Table S2**). As shown in **Figure 7A**, the expression of *CsKFB* genes was significantly increased in the *AtCYP79B2* overexpression lines compared with that of wild-type plants.

## Discussion

### Overexpression of *AtCYP79B2* Increases Auxin Contents But Does Not Affect Indole Glucosinolate Production in Camelina

IAA a major auxin in plants is made mainly through the YUC pathway, the common auxin biosynthesis pathway. The YUC pathway is a simple two-step biosynthesis pathway using two enzymes, TAA (Tryptophan Aminotransferase of Arabidopsis) and YUCCA (Flavin containing monooxygenase) (Bak and Feyereisen, 2001; Kim et al., 2007; Dai et al., 2013; Korasick et al., 2013). In addition to the YUC pathway, IAA can be made from IAOx in Arabidopsis under stress conditions (Zhao et al., 2002; Nonhebel et al., 2011). Overexpression of *CYP79B2*-encoding IAOx producing enzyme, or disruption of the IAOx-consuming enzyme CYP83B1 results in the increased levels of IAA in Arabidopsis, indicating the production of IAA from IAOx (Delarue et al., 1998; Barlier et al., 2000; Zhao et al., 2002; Sugawara et al., 2009). Our data suggest that the IAOx-dependent IAA biosynthesis pathway is conserved in Camelina as well. It was previously shown that *CsCYP79Bs*, *Csa11g003080*, and *Csa10g002730* were coexpressed with other genes encoding auxin transporters and auxin biosynthesis enzymes in response to stresses (Heydarian et al., 2018). It is possible that the IAOx-derived IAA production in Camelina is relevant to stress-induced growth modulation as shown in Arabidopsis (Franklin et al., 2011).

Previous studies have shown that Arabidopsis plants overexpressing *CYP79B* contain increased indole glucosinolates (Zhao et al., 2002). However, we were not able to detect indole glucosinolates in young seedlings or old leaves of both Camelina wild type and all three overexpression lines (**Figure 2B**). Over 30 different structures of glucosinolates are found in Arabidopsis and their profiles vary depending on organs and ecotypes (Kliebenstein et al., 2001). *Camelina* seeds accumulate aliphatic glucosinolates including glucoarabin (9-(methylsulfinyl)nonylglucosinolate), glucocamelinin (10-(methylsulfinyl)decylglucosinolate), and 11-(methylsulfinyl)undecylglucosinolate (Berhow et al., 2013; Das et al., 2014; Russo et al., 2014). The *C. sativa* genome study identified that the *C. sativa* genome represents a whole-genome triplication event relative to the *Arabidopsis thaliana* genome (Kagale et al., 2014). Indeed, we found that CYP79Bs and KFBs of Arabidopsis and Camelina have high sequence identities (**Table 1**; **Table S2**). But, the glucosinolate profiles in Camelina are quite different from those of Arabidopsis. It is possible that other factors such as specific pest infestation or pathogen attack are required to activate glucosinolate production. Apparently, IAOx is not a limiting factor for Camelina to produce indole glucosinolates, at least in seedlings and leaves.

### Altered IAOx Metabolism Affects Phenylpropanoid Production Through Transcriptional Activation of Genes Functioning in PAL Degradation in Camelina

The fact that the expression of *PALs* is not altered while these *CsKFB* -like genes are up-regulated in the *AtCYP79B2* overexpression lines suggests that increased *CsKFB* expression can contribute to reduced PAL activity as well as the reduction of phenylpropanoids in the transgenic lines. The activation of *KFB* expression and reduced PAL activity in the Camelina transgenic lines are reminiscent of a molecular mechanism of phenylpropanoid repression in Arabidopsis high-aldoxime mutants (Kim et al., 2019). The biosynthetic pathway of phenylpropanoids and its regulation have been extensively studied. Studies have revealed that the phenylpropanoid pathway can be regulated at various levels. Transcription factors or general transcription machineries such as Mediator subunit 5 (MED5) are involved in the expression of genes encoding biosynthetic enzymes (Bonawitz et al., 2012; Dolan et al., 2017). Feedforward or feedback regulations and posttranslational modification of enzymes such as phosphorylation or ubiquitination play roles in the activity or stability of enzymes (Zhang et al., 2013; Zhang et al., 2015). In addition, the protein-protein interaction is critical to form the lignin metabolon (Gou et al., 2018). Our data presented here and the previous Arabidopsis studies indicate that phenylpropanoid metabolism can be affected by the status of other metabolite biosynthetic pathways (**Figure 7B**). Since PAL functions at the entry point of the phenylpropanoid pathway, the influence of aldoxime metabolism on PAL stability can affect the entire phenylpropanoid production. It appears that the molecular mechanism behind the link between aldoxime metabolism and phenylpropanoid production is conserved between Arabidopsis and Camelina. It was shown that the content of glucosinolates is inversely correlated with the level of sinapine (Russo and Reggiani, 2012). It is possible that the negative correlation between the sinapine content and the glucosinolate content in Camelina may be explained by the phenylpropanoid repression by aldoxime metabolism. It is worth noting that Arabidopsis produces indole glucosinolates from IAOx but Camelina does not although both produce IAA and camalexin from IAOx. This result further confirms that aldoximes or their derivatives, not glucosinolates, are key molecules affecting transcriptional activation of KFBs. Recent studies have identified aldoxime-producing enzymes in various species including monocots and gymnosperms (Irmisch et al., 2013; Irmisch et al., 2015; Luck et al., 2017; Bhalla et al., 2018; Dhandapani et al., 2019). Considering that aldoxime metabolism is widely spread in the plant kingdom, this type of link may exist in species beyond Brassicales.

## Data Availability Statement

Sequence of genes and proteins in this study can be found in the GenBank under the following accession numbers: AtCYP79B2 (At4g39950), AtCYP79B3 (At2g22330), AtKFB1 (At1g15670), AtKFB20 (At1g80440), AtKFB39 (At2g44130), AtKFB50 (At3g59940), Csa10g002730 (XP_019086033), Csa12g003200 (XP_010446588), Csa12g002890 (XP_010446562), Csa11g003080 (XP_010437120), Csa7g052430 (XP_010416994), Csa16g044070 (XP_010472222), Csa9g086670 (XP_010429154), Csa06g050450 (XP_010518004), Csa05g006160 (XP_010508332), Csa04g061960 (XP_010506327), Csa07g005130 (XP_010413616), Csa05g091560 (XP_010512288), Csa16g006020 (XP_010469249), Csa09g094570 (XP_010417707), Csa07g059430 (XP_010429956), Csa17g021220 (XP_010476655), Csa03g019430 (XP_010497299), Csa14g018700 (XP_010459104), Csa06g039710 (XP_010516949), Csa05g016810 (XP_010509401), Csa04g050600 (XP_010505261), Csa06g027730 (XP_010515816), Csa04g039260 (XP_010504083), Csa09g061550 (XP_010426963), Csa01g011180 (XP_010482945), Csa15g012820 (XP_010464706).

## Author Contributions

DZ and JK conceived and designed the experiments. DZ, YS, and RD performed the experiments. TL generated phylogenetic trees. DZ and JK wrote the manuscript. All authors read and approved the final manuscript.

## Funding

This work was supported by USDA NIFA Hatch (005681) and a startup fund from the Horticultural Sciences Department and Institute of Food and Agricultural Sciences at the University of Florida. This work was also supported by the Principal Investigator of Postdoctoral Fellowship Program through the National Research Foundation of Korea (NRF) grant funded by the Ministry of Education (No.2018R1A6A3A03013074, YS, Republic of Korea).

## Conflict of Interest

The authors declare that the research was conducted in the absence of any commercial or financial relationships that could be construed as a potential conflict of interest.
