# Supplementary material for: Aldoxime Metabolism Is Linked to Phenylpropanoid Production in Camelina sativa
Source: Front Plant Sci. 2020 Feb 5;11:17. doi: 10.3389/fpls.2020.00017 (PMC7025560; doi:10.3389/fpls.2020.00017)
Supplement: Supplementary file 1 [file DataSheet_1.doc]

Supplementary Material

**
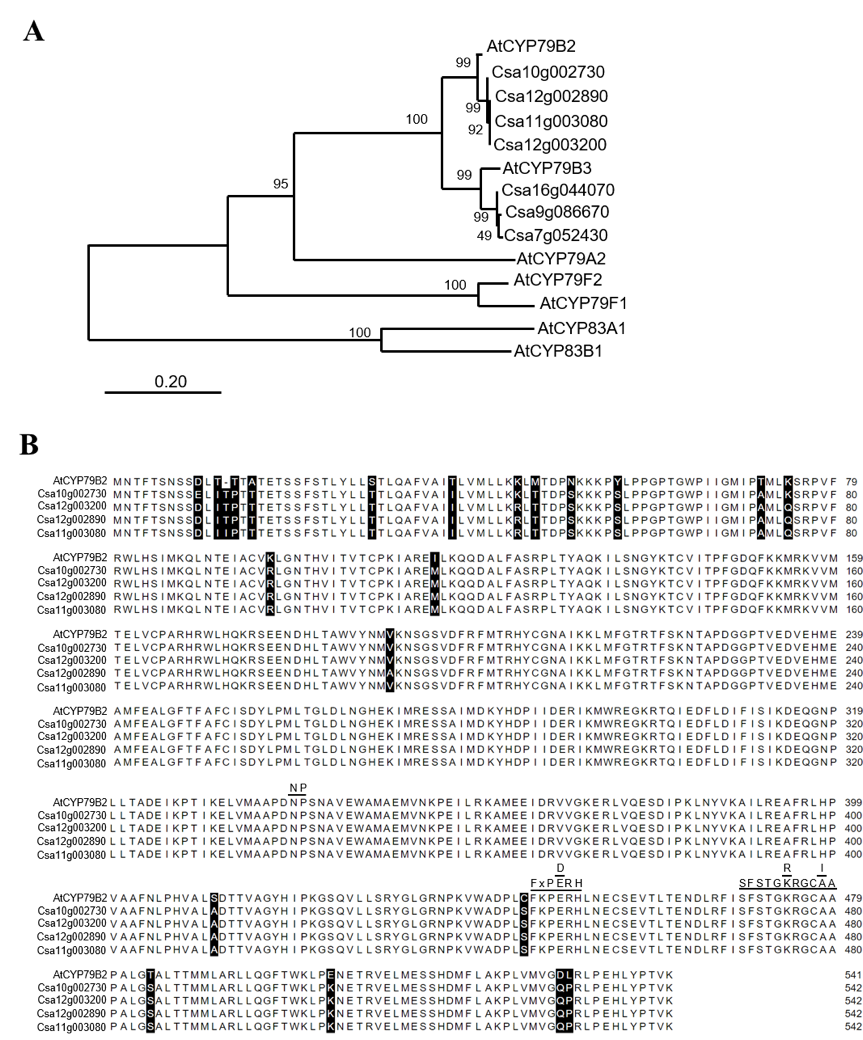
**

**Supplementary Figure 1.** A phylogenetic tree of CsCYP79Bs and AtCYP79B family.

(A) Phylogenetic tree of potential CY79Bs in Camelina*.* The tree was constructed with Maximum Likelihood method and Poisson correction model. Bootstrap with 1000 replicates was performed. (B) Alignment of AtCYP79B2 and its homologs from Camelina. Conserved motifs for CYP79 family protein were labeled with black lines.

**
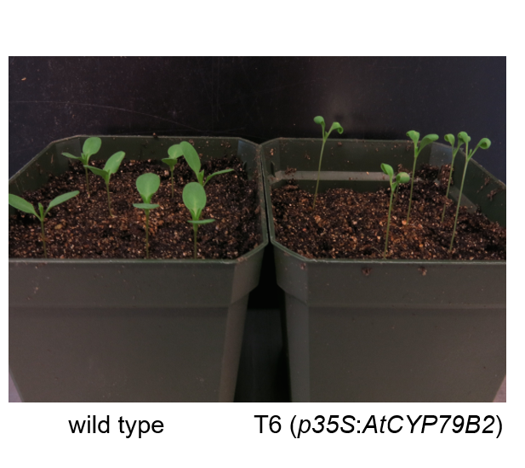
**

**Supplementary Figure 2.** Soil-grown Camelina plants overexpressing AtCYP79B2 display morphological phenotypes distinguishable from wild type.


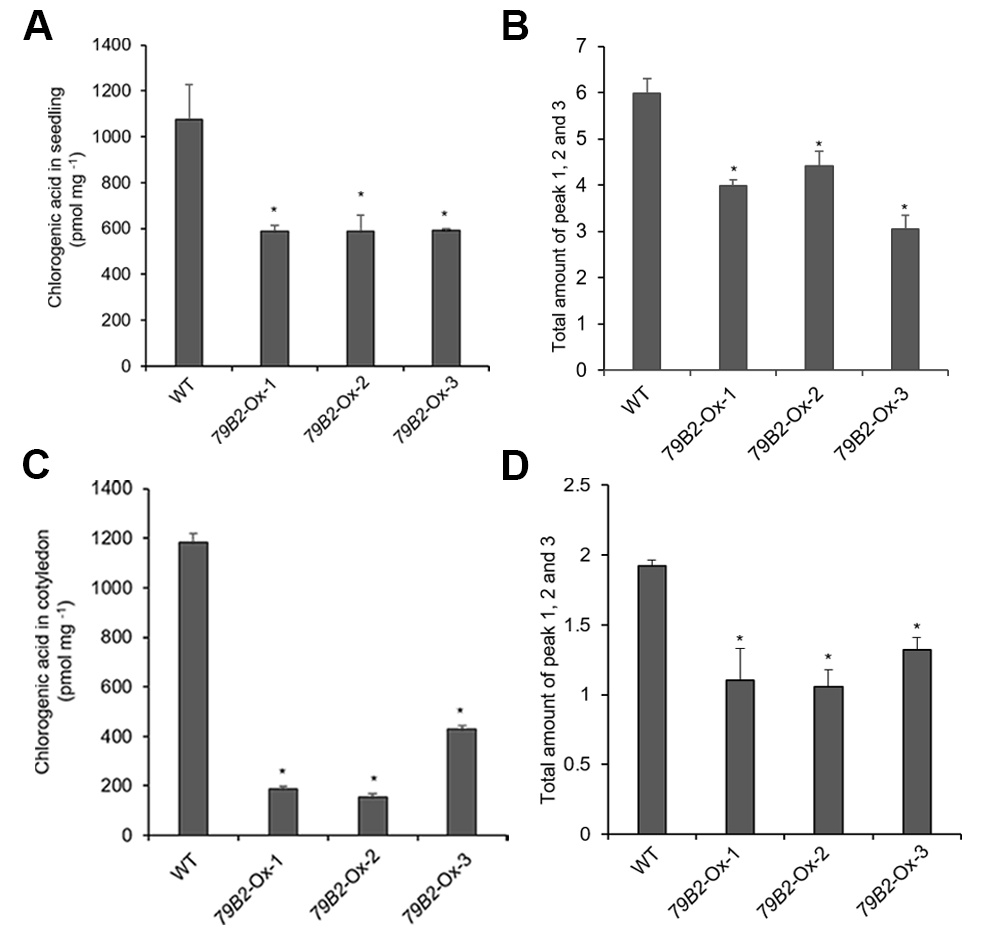


**Supplementary Figure 3. Phenylpropanoid contents in seedlings and cotyledons.**

Chlorogenic acid contents and the total sum of peak 1, 2 and 3 in seedlings (A, B) and cotyledons (C, D) of the *AtCYP79* overexpression lines compared with wild type are shown. Cotyledon and leaf samples were collected from 2-week-old soil grown plants and seedlings were grown on MS plates for 7 days. Data represent mean ± SE from three independent biological replicates. * represents *P* < 0.05 (two-tailed student’s t-test) when compared with wild type.

**Supplementary Table 1.** A Diagnostic MRM transitions and optimized instrument setting for IAA quantification

| Compound | MRM transition | CE (V) | RF (V) | Retention time (min) | LOD (fmol) | Linear range (pmol)/R^2^ |
| --- | --- | --- | --- | --- | --- | --- |
| IAA | 175.983 > 130.071  > 103.071  > 77.000 | 17.258  33.138  45.101 | 47.237 | 3.65 ± 0.051 | 5.0 | 0.01-500/0.9999 |
| [^13^C_6_]-IAA | 181.933 > 136.000  > 109.000  > 81.000 | 16.710  33.012  43.248 | 49.461 |

**Supplementary Table 2.** Sequence identities among Arabidopsis KFBs and Camelina KFBs

| Group | Gene name | AtKFB1 (%) | AtKFB20 (%) | AtKFB39 (%) | AtKFB50 (%) |
| --- | --- | --- | --- | --- | --- |
| CsKFB1-like | Csa17g021220 | 89 | 70 | 36 | 34 |
|  | Csa03g019430 | 88 | 68 | 35 | 34 |
|  | Csa14g018700 | 87 | 66 | 36 | 34 |
| CsKFB20-like | Csa09g094570 | 67 | 81 | 31 | 33 |
|  | Csa07g059430 | 67 | 83 | 31 | 34 |
| CsKFB39-like | Csa06g050450 | 39 | 37 | 79 | 63 |
|  | Csa05g006160 | 37 | 36 | 78 | 62 |
|  | Csa04g061960 | 38 | 36 | 77 | 61 |
| CsKFB50-like | Csa07g005130 | 37 | 35 | 59 | 85 |
|  | Csa05g091560 | 36 | 36 | 60 | 86 |
|  | Csa16g006020 | 35 | 34 | 59 | 85 |

**Supplementary Table 3.** Transcript abundance of *CsKFBs* in Camelina wild type.

| Gene | Ct value (mean ± sd) |
| --- | --- |
| CsKFB1-like | 24.06 ± 0.257829 |
| CsKFB20-like | 23.34 ± 0.298016 |
| CsKFB39-like | 26.47 ± 0.122349 |
| CsKFB50-like | 21.65 ± 0.202467 |

**Supplementary Table 4.** The list of primers used for this study

| Primer Name | Sequence (5’ -> 3’) |
| --- | --- |
| P1 | ACGAACAAGGCAACCCATTGCTTAC |
| P2 | GGGATGTCGGATTCTTGAACGAGTCTC |
| P3 | ACA ATT TCC CGC TCT GCT GTT GTG |
| P4 | AGG GTT TCT CTC TTC CAC ATG CCA |
| P9 | GATGTGTCTACGTGGCAGTGGG |
| P10 | CACTTTCTTCCACGTGTCGTTCGT |
| P19 | GGGGACAAGTTTGTACAAAAAAG  CAGGCTTCATGGTAGTGTCAAAGAAGAAATC |
| P20 | GGGGACCACTTTGTACAAGAAAGC  TGGGTTTTTAGACATAGATTGAAGAATAAGAAAAAG |
| P47 | TGGTGGAGCCTTACAACGCTACTT |
| P48 | TTCACAGCAAGCTTACGGAGGTCA |
| P98 | ACGTGGCATAAAGTTGGGGAG |
| P99 | GAAGCAACCAGCTTGAACGTG |
| P100 | GAAGACGGGAAGTTCAGTTTTCGC |
| P101 | GGAAGAGTAGAGAAAGCTGAAGGTAT |
| P102 | GTTATGGAGACGATTAGGCTCCC |
| P103 | GAAGACTCGAAAAACCAGAGGGAG |
| P121 | GCTTCACGAGATGGATCCGTTAC |
| P122 | GAGTTGATCTCACGCTCGAT |
| P125 | GAGTCGGAGCTGAGATTGCTTC |
| P126 | GTTTTCCATCAGAAATCGCAGAC |
